# Prevalence and perception of drug use amongst secondary school students in two local government areas of Lagos State, Nigeria

**DOI:** 10.4102/sajpsychiatry.v26i0.1428

**Published:** 2020-07-28

**Authors:** Rebecca O. Soremekun, Bukola O. Folorunso, Oluwatosin C. Adeyemi

**Affiliations:** 1 Department of Clinical Pharmacy and Biopharmacy, Faculty of Pharmacy, University of Lagos, Lagos, Nigeria; 2 HealthMaps Pharmacy, Lagos, Nigeria

**Keywords:** drug abuse, prevalence, youth, alcohol, opioid, non-medical drug use

## Abstract

**Background:**

Drug abuse, an excessive and persistent self-administration of a drug without regard to the medically or culturally accepted patterns, has been reported amongst teenagers and adolescents in various regions of the world.

**Aim:**

This study aimed to measure the prevalence of drug use amongst students of junior and senior secondary schools (aged 10–15 years).

**Setting:**

This study was conducted at two local government areas in Lagos State.

**Methods:**

The cross-sectional study was carried out in Ikotun or Igando local council development area (LCDA) and Ikoyi LCDA of Lagos State. Students were sampled using stratified random sampling with classes as strata and sampling performed by balloting. The modified WHO Model Drug Use Survey Questionnaire was distributed to the students for self-reporting. Ethical approval was received from district school boards.

**Results:**

A total of 1048 students participated in the survey. In this study, alcohol had the highest lifetime drug prevalence rate (29.1%), followed by pharmaceutical opioids (9%). Gender, educational level, type of school management, and geographical economic distribution were found to be predictors of prevalence of drug use. This study demonstrated significant differences in the prevalence of tobacco and opioids use among students in private and public schools; and documented statistically significant differences in the prevalence of cocaine use between low income and high-income areas in two LCDAs in Lagos, Nigeria.

**Conclusion:**

Prevalence of lifetime, recent use, and current use of drugs among secondary school students in two LCDAs located in Lagos State, Nigeria were documented with alcohol as the drug with the highest prevalence.

## Introduction

Drug abuse, an excessive and persistent self-administration of a drug without regard to the medically or culturally accepted patterns, is a major public health problem all over the world.^1^ It could also be viewed as the use of a drug to the extent that it interferes with the health and social function of an individual.^1^ The World Health Organization (WHO) identifies abuse and the use of drugs by adolescents as one of the most disturbing health-related phenomena around the world. Several adolescents of school age (10–21 years) have been known to experience mental health problems, which may be transient or sustained and may adversely affect their learning and development.^2^

In Nigeria, common drugs of use were categorised by Haladu^3^ as social drugs, stimulants hallucinogens, narcotics sedatives, tranquilisers and miscellaneous solvents. Haladu^3^ also identified common factors motivating students to start drug use, including experimental curiosity, peer influence, lack of parental supervision, personality problems, the need for energy to work for long hours, availability of drugs of use, exposure on social media, the need to prevent the occurrence of withdrawal symptoms, purchasing power and cultism. These factors have been confirmed in several findings amongst Nigeria adolescents. Students, especially those in secondary schools, tend to see the drug user as one who is tough, bold and strong.^4^ Many adolescents have been known to use drugs at the instance of peers, elders or siblings and those who usually feel inadequate have been known to use drugs to achieve social acceptance.^5^ Some Nigerian secondary school adolescents under the influence of Indian hemp have been reported to shed all inhibitions and produce behaviour that is inconsistent with school discipline.^5^ Students with poor academic records, a history of instability and family or social problems, have been found to use the drugs that are supposed to improve wakefulness, whilst others use drugs to increase their self-confidence, heighten pleasure, cope with feelings of depression, inadequacy and facilitate communication.^6,7,8^

According to the U.S. Department of Health and Human Service, there was a drop in the use of drugs especially tobacco, alcohol and marijuana during the late 1980s. However, a recent upturn has been reported with adolescents beginning experimentation with psychoactive drugs as early as the 8th-grade equivalent to Junior Secondary School 2 in Nigeria.^9^

A few surveys have reported substance use amongst secondary school students (aged between 10 and 15 years) in two other regions in Nigeria^8,10^ and the recent BBC report on substance use in the Northern region of the country reveals a high level of use amongst youths.^11^ However, the Nigerian Drug Use survey report released in 2018 did not capture use amongst secondary school students leaving a gap in the country’s drug use data.^12^ It is therefore important to identify specific drug use patterns amongst this category of people so that appropriate interventions can be planned for and implemented.

This study was therefore carried out in Lagos, the commercial capital of the country to contribute data to fill this gap. The study set out to determine the prevalence of drug use amongst secondary school students, elicit their perception of drug use in their environment and identify the factors that may contribute to the initiation of drug use amongst this category of students within the city. It also contributes data on the differences between the affluent and low per capita neighbourhoods which before now was not available in the literature.

## Method

The study, a comparative cross-sectional survey using the WHO model drug use survey questionnaire,^13^ was carried out amongst secondary school students in two local council development areas (LCDAs) – Ikotun/ Igando, a low per capita neighbourhood, and Ikoyi, a high per capita neighbourhood in Lagos State.^14^ A total of 10 secondary schools (made up of senior and junior secondary schools) were included as sites for the survey made up of

- public schools: three secondary schools (made up of three senior and three junior secondary division) in Ikotun or Igando LCDA and three secondary schools (made up of three senior and three junior secondary division) in Ikoyi LCDA
- private schools: three secondary schools (made up of three senior and three junior secondary division) in Ikotun or Igando LCDA and one secondary school (made up of one senior and one junior secondary divisions) in Ikoyi LCDA.

A pilot study was carried out using 10 students in Ikotun or Igando LCDA to pre-test the questionnaire, following which some modifications were made to simplify some words to match the students’ reading level.

The sample size was calculated to be 368 using the Fisher formula.^15^ However, 1078 students were sampled to increase the reliability of finding differences in the population under study. With more students attending the public schools to the private schools, the sample was shared proportionally.

All eligible students were presented with information about the study as a group in a classroom, and those willing to participate were then asked to present in a designated classroom. Questionnaires were self-administered and retrieved as soon as possible. The students were assured of confidentiality, and their teachers were not in the same room during the administration of the questionnaire. Data were collected over 2 months, summarised using appropriate descriptive statistics and tests of association determined using the chi-square test. The significance level was set at 95%.

Prevalence was reported in four categories as follows:

- Lifetime prevalence was measured by respondent’s self-report of any drug use at any point in their lifetime.
- Infrequent use or 12 months prevalence refers to the percentage of students who have taken the drug in question in the last 12 months preceding the study.
- Recent use or 1-month prevalence refers to the percentage of students who have taken the drug in question in the last 30 days preceding the study.
- Current use or 1-week prevalence was measured by respondent’s self-report of drug use at any point in the last 7 days.

For medicinal opioids, non-medical drug use was differentiated from medical use by asking participants to only report use without a prescription.

### Ethical consideration

Ethical approval was obtained from the education district office Agege and Eti-Osa Education District Boards. The authors ensured quality and integrity of the survey and sought informed consent (for students under the age of 18 years, the teachers gave consent but student assent was sought). Data collected were anonymised to ensure that no identifying information was collected. All participants participated voluntarily. Due care was taken to ensure that there was no injury to participants, but information was given to all participants to ensure that any one injured is able to seek redress through the Approving Education Office and the Research Ethics Committee of the Post-Graduate College of Pharmacy (Reference number: EDI/155/003/141). This research was performed independently by the researchers and there is no conflict of interests to report.

## Result

The number of male students who participated in the study was 533 (50.86%), whilst female students were 515 (49.14%). The age bracket with the highest frequency was 15–16 years, constituting 40.17%. Sixteen students did not disclose their age (Table 1).

**TABLE 1:** Age distribution of respondents in the two local council development areas of Lagos State (*n* = 1048).

Age	Frequency	Percentage
10 years old or less	1	0.10
11–12 years	62	5.92
13–14 years	379	36.16
15–16 years	421	40.17
17–18 years	150	14.31
19 years old or more	19	1.81
Undisclosed	16	1.53

**Total**	**1048**	**100.0**

Note: Secondary school students reported age in a range.

In total, 1048 respondents participated in this study (97.2% response rate) of which junior secondary school students who participated in the study were 500 (47.71%) and senior secondary school students numbered 548 (52.29%).

Majority of the respondents were in public schools 73% (*n* = 765), whilst 27% (*n* = 283) attended private schools.

The percentage of students living in low-income areas was 64.5% (*n* = 676), whilst those living in high-income areas made up 35.5% (*n* = 372).

The highest current use and lifetime prevalence by percentage were seen amongst alcohol users. Other current user and lifetime prevalence for substances are presented in Table 2.

**TABLE 2:** Drug use prevalence (%) amongst secondary school students in Lagos State, Nigeria (*n* = 1048).

	Prevalence rates	Tobacco (%)	Alcohol (%)	Cannabis (%)	Cocaine (%)	Inhaled things (%)	Tranquilisers (%)	Sedatives (%)	Heroin (%)	Opioids (pharmaceutical) (%)
**Population**	Current user	1.5	11.6	1	0.4	2	2.3	1.4	1.3	3.2
	Lifetime prevalence	4.1	40.3	2.4	1.4	5.6	6	3.9	3.5	13.4
**School management**										
Public school	Current user	1.5	9.2	1	0.4	1.34	1.8	1.2	1.1	2.7
	Lifetime prevalence	3.7	29.1	2	1.3	4.2	4	3	2.6	9
Private school	Current user	0	2.5	0	0	0.7	0.5	0.3	0.3	0.6
	Lifetime prevalence	0.3	11.2	0.4	0.1	1.4	2	1	1	4.4
**Gender**										
Male	Current user	0.4	7.1	0.9	0.3	1.5	1.2	1.2	1.2	2.3
	Lifetime prevalence	3.4	23.5	2.2	1.1	4.4	3.5	2.7	2.7	7.7
Female	Current user	0.2	4.6	0.1	0.1	0.5	1.2	1.2	1.2	2.3
	Lifetime prevalence	0.7	16.8	0.2	0.4	1.2	2.5	1.2	0.9	5.6
**School level**										
Junior secondary school	Current user	0.2	3.9	0.3	0.3	1	0.8	0.1	0.4	1
	Lifetime prevalence	0.3	15.5	0.5	0.1	2.2	1.8	0.4	1.4	4.7
Senior secondary school	Current user	1.3	7.1	0.7	0.7	1.1	1.5	1.3	1	2.3
	Lifetime prevalence	3.8	24.8	1.9	1.3	3.4	4.2	3.5	2.1	8.7
**Economic level**										
Low-income area	Current user	0.6	7.4	0.2	0	1.2	1.2	1.1	0.5	1.6
	Lifetime prevalence	1.9	25.2	0.8	0.3	3.5	3.5	2.7	2.1	8.5
High-income area	Current user	1	4.3	0.8	0.4	0.8	1.2	0.4	0.9	1.6
	Lifetime prevalence	2.2	15.1	1.6	1.2	2.1	2.5	1.2	1.4	4.9

Note: Current user prevalence – respondents who report using drug within the last 7 days; lifetime prevalence – respondents’ self-report of drug at any point in their lifetime.

Most secondary school students in the two LCDAs sampled initiated non-medical drug use at age 13–14 years (Table 3). The most common drug to which the students were introduced was alcohol, followed by the pharmaceutical opioids at age 13–14 years. However, students reported being introduced to non-medical drug use at 10 years or less.

**TABLE 3:** Age at which secondary school students recollect using drug for the first time (non-medical).

Age Drug used	10 years or less (%)	11–12 years (%)	13–14 years (%)	15–16 years (%)	17–18 years (%)	19 years or more (%)
Tobacco	0.95	0.57	0.67	1.15	0.57	0.38
Alcohol	7.63	7.63	13.26	8.68	2.58	0.48
Cannabis	0.48	0.48	0.38	0.38	0.38	0.29
Cocaine	0.38	0.29	0.10	0.29	0.29	0.15
Inhaled things	0.95	1.43	1.24	1.05	0.67	0.15
Tranquilisers	0.86	0.67	1.81	1.72	0.29	0.48
Sedatives	0.38	0.86	1.05	1.15	0.29	0.19
Heroin	0.38	0.76	0.76	0.57	0.76	0.29
Opioids	2.19	2.86	3.82	2.48	1.72	0.19

Note: (%) – percentage of students with initial drug use at this age; age – as reported (in ranges); drug used – only non-medical use of drug is reported.

Students in this survey most commonly identified family members (17.4%) as those who introduced them to drug use, followed by friends (6.1%). Other individuals who were identified include casual acquaintances (2.3%), classmates (2.2%), others (1.9%) and drug pushers (1.2%) in that order. However, a significant number of students did not remember the person who introduced them to drug use.

Some of the students who had ever used drugs without a medical prescription identified ‘treatment of health disorder’ as the most important perceived reason for the use of drugs, whilst ‘relief of stress’ was the next most important reason for use of drugs. Other reasons perceived to be important by students include ‘need to improve work’, ‘curiosity’, ‘enhancement of sex’, ‘enjoyment’, ‘religious custom’, ‘social acceptance’, ‘sociability’ and ‘others’ in that order. However, a large percentage (66.89%) could not identify any specific need for the use.

A few respondents considered it very easy to get Marijuana (cannabis) (11.67%) and pharmaceutical opioids (tramadol, codeine) (18.13%). By percentages, students in lower economic areas (10.69%) reportedly thought that it was easier to obtain cannabis than their counterparts in higher economic areas (7.44%). There was no significant difference in the number of students who perceived it easy to get opioids in both low (5.36%) and high economic settings (5.82%) surveyed.

Senior secondary schools had higher prevalence of students abusing tobacco (*X*^2^ = 30.6, *p* < 0.0001), alcohol (*X*^2^ = 25.9, *p* < 0.0001), cannabis (*X*^2^ = 8.4, *p* = 0.038), cocaine (*X*^2^ = 10.4, *p* = 0.015) and pharmaceutical opioids (codeine and tramadol) (*X*^2^ = 13, *p* = 0.005) when compared to students attending junior secondary school in the same LCDAs in Lagos.

Secondary schools (senior and junior) had higher prevalence of male students abusing tobacco (*X*^2^ = 30.6, *p* < 0.0001), alcohol (*X*^2^ = 25.9, *p* < 0.0001), cannabis (*X*^2^ = 8.4, *p* = 0.038), cocaine (*X*^2^ = 10.4, *p* = 0.015) and pharmaceutical opioids (codeine and tramadol) (*X*^2^ = 13, *p* = 0.005) when compared to female students attending secondary school (senior and junior) in the same LCDAs in Lagos.

The prevalence of cannabis and cocaine use amongst secondary school students (senior and junior) living in lower economic areas was higher (cannabis use [*X*^2^ = 14.8, *p* = 0.002] and cocaine use [*X*^2^ = 14.6, *p* = 0.002]) than amongst those in higher economic areas in Lagos. However, the prevalence of tobacco and pharmaceutical opioids use amongst secondary school students (senior and junior) was higher (tobacco (*X*^2^ = 8.22, *p* = 0.042) and pharmaceutical opioids (*X*^2^ = 14.8, *p* = 0.002)) amongst students attending public schools than amongst private school students.

Students who thought that cannabis was accessible (*X*^2^ = 79.4, *p* < 0.0001) were likely to be from schools located in higher economic areas. This relationship was not apparent with the other drugs of abuse.

A quarter of the respondents claimed to have taken drugs not listed in the questionnaire. Some of them reported inhaling or smoking things such as perfume, gum, ammonia (NH3), spirit and spray glue. Other pharmaceutical drugs were also reported including chlorpheniramine and pain relievers such as diclofenac and aspirin.

## Discussion

This study identified lifetime, recent use and current use prevalence of tobacco, alcohol, cannabis, cocaine, inhaled substances, tranquilisers, sedatives, heroin and pharmaceutical opioids amongst secondary students in public and private schools. The highest current use and lifetime prevalence by percentage were seen amongst alcohol users and most of the students in the two LCDAs initiated non-medical drug use at age 13–14 years.

### Demography

This study captured data from respondents aged 10–14 years, contributing to the body of knowledge about this particular demographic class. The mean age (15–16 years) for secondary school students reported in this study is comparable to 15.9 years reported in the study by Oshodi et al. who used a similar method (and tool).^16^ The age range is consistent with 15–17 years reported by the Federal Ministry of Education in a demographic report of secondary school students in Nigeria.^17^

### Prevalence rates

The studies by Oshodi et al.^16^ and Adebowale et al.^18^ reported a lower lifetime prevalence for ‘gateway’ drug, and then alcohol was found in this study. The higher prevalence recorded here (40.3%) may be because of the recent introduction of unregulated locally brewed alcoholic concoctions containing herbal extracts in smaller unit packs into the Nigerian market. These packs are convenient to hide on young persons.^19^ There have been calls by stakeholders and non-government organisations (NGOs) for a blanket ban on alcohol produced in sachets and small plastic bottles made available without an age restriction on the streets.^20^

Tobacco lifetime prevalence (5.2%) amongst secondary school students in Lagos as reported by Oshodi et al.^16^ was higher than the level reported in this study (4.1%). This suggests a possible trend of decreasing tobacco use amongst young people and warrants further investigation. Contrariwise, a recent national survey did not reflect similar outcomes in the general population of 15–64 year olds, and hence the observed downward trend may be unique to this age group.^7^

The reported incidence of cocaine and heroin use in this demography has remained unchanged from the levels reported by Oshodi et al.^16^ There seems to be no concerted or effective regulatory effort tailored to this demography to reduce the use of these two drugs. The most recent initiative funded by the National Agency for Food and Drug Administration and Control (NAFDAC) and implemented by the not-for-profit, Young Pharmacists’ Group, Youth Against Drug Use (YADA) took off in 2018. The YADA project specifically targets in-school youth with interventions to prevent, reduce and stop the initiation of drug use in all local government areas within Nigeria.^21^ There have been no published data yet, hence the impact of the project in the last year cannot be measured.

The lifetime use prevalence of cannabis in this study, though lower than that seen in an earlier study,^22^ does not appear to be directly because of any effect of regulation as no programme can be directly traced to reduce the use of the drug in this demography. Of particular note is the fact that the use of cannabis is even currently more widespread amongst the adult population, with Nigeria reporting one of the highest local seizures of the drug in 2016.^7^ The recent national survey on drug use reports a higher prevalence of cannabis use amongst the general population than in this survey.^7^

The prevalence of the use of inhaled substances (5.6%) was higher in this study than that reported amongst the general population in a recent survey,^7^ but in the survey carried out by Oshodi et al.,^16^ the reported lifetime and current user prevalence rates were comparable. The age of initiation in the national survey was similar to the age of initiation reported in this study.

The national survey also reported a lower prevalence of non-medical use of sedatives and tranquilisers than identified in this study.^7^ However, Oshodi et al.^16^ reported comparable lifetime and current user prevalence rates.

### Contributory factors

Gender has been reported as a significant factor in the consumption of drugs.^16^ The prevalence rates for the use of tobacco, alcohol, cannabis, inhaled substances and sedatives were significantly higher in men than in women in this study. These data are supported by the recent general population drug use survey.^7^

This study also identified a higher prevalence of tobacco, alcohol, cannabis, cocaine, tranquilisers, sedatives and opioids (tramadol and codeine) amongst senior secondary school students than in the junior secondary school students. There were no prior studies powered to identify these differences, and hence more studies are required to test this difference.

Similarly, the study also identified an increased prevalence of drug use with increasing age. This may be because of unmitigated initiation of drug use at a young age, followed by persistence in use compounded by increasing peer influence. As identified in several studies, family members and peers are an important factor in the initiation and persistence of drug use.^23^

This study identified a higher likelihood for a higher lifetime and current user prevalence of tobacco and pharmaceutical opioid use amongst public secondary school students compared to private secondary schools in Lagos State. However, this does not hold for other drugs of use surveyed. This may be related to insufficient supervision for students in public schools, and other attendant socio-economic problems (such as overcrowded classes, absenteeism, increased health risk and violent confrontations outside the school premises) that are replete amongst students attending public schools in Nigeria who are mainly from the lower economic strata.^24^

Interventions to reverse this trend must be holistic and take into cognisance the socio-economic background of the student.

Per capita income is also a significant factor. Students attending schools in areas populated by people with higher per capita income were more likely to be abusing cannabis and cocaine than those in areas with lower per capita income. This may be related to the higher purchasing power available to students in these areas. Therefore, interventions to ameliorate the uptake of cannabis and cocaine amongst students in higher per capita income areas must take into cognisance this important contributing factor.^25^

In this study, students’ perception of ease of access to drugs did not correlate to the use of drugs as postulated by Ndom and Adelekan.^26^ Students who expressed their perception that it was easy to get drugs were not more likely to be currently using drugs than those who thought otherwise.

Reasons for using drugs reported by some of the students include relief of stress, treatment of an illness and performance enhancement. These are supported by findings from earlier surveys.^22,26^

## Study limitations

The sample under study may not adequately represent the population of secondary school students in Lagos State, even though the effort was made to ensure that the study recruited as widely as possible. This study also accounted for adolescents and young adults in school and did not take into consideration peers who may be out of school. These limit the generalisation of this study’s results.

## Conclusion

This study documented lifetime, recent use and current use prevalence of tobacco, alcohol, cannabis, cocaine, inhaled substances, tranquilisers, sedatives, heroin and pharmaceutical opioids amongst secondary school students in two LCDAs located in Lagos State, Nigeria.

Alcohol was the drug with the highest prevalence amongst the students followed by the opioids (tramadol and codeine), whilst cocaine had the lowest prevalence. The prevalence of cigarette was low in comparison with the other social drug – alcohol.

Age, sex, educational level, type of school management and the economic area where the school is located all contribute significantly to the prevalence of drug abuse amongst secondary school students in Lagos.

The perception of ease of access to drugs was not significantly associated with drug use or the economic level of the area where the school is located.

Students identified stress, medication for health reasons and performance enhancement as reasons for the use of drugs.
